# Texas Surgery

**Published:** 1886-04

**Authors:** 


### EDITORIAL NOTES.

### TEXAS SURGERY.

We have been favored by the Chairman of the Special Committee on Surgery, (Dr. Geo. Cupples) appointed by the Texas State Medical Association, with advance sheets of their report, which is to be submitted at the Dalllas convention: The resolution under which this special committee was appointed, contemplated the compilation of all surgical operations performed by Texas Surgeons in Texas up to the present time, to be reported to the association. This then would be a basis upon which yearly reports are to be made, and thus a record of Texas Surgery can be kept, but it is impossible ever to collate a complete record. It is a good example to other States, and if followed, the American Medical Association can be put in possession of valuable clinical data in no other way attainable. In 1878 a report was made up to that date, showing some 1875 operations. The present report brings the subject up to the present date, and covers a grand total as follows:

| | | *Deaths.* | *Death Rate.* |
|---|---|---|---|
| Major operations | 2080. | 331. | 15.91 |
| Minor operation | 2213. | 19. | 0.85 |
| Grand total | 4293. | 350. | 8.15 |

There are various opinions in the Texas profession as to the utility of such a report; and we know of our own knowledge, that there are many surgeons who have not reported any of their operations, and who cannot be induced to do so, believing that it is impossible to procure a complete and *reliable* record. Thus, the record must ever be incomplete, and of only relative value. But the cases reported make a very creditable showing.

The chairman of the committee deserves credit for his zeal and labor in the cause, and they are worthy of a better response on the part of the profession of the State. In the report accompanying the tables of operations, the chairman says:

"If the whole truth must be told, the writer of this report remembers to have read in the London Lancet some years ago, "what good (professionally, that is) can come out of Texas?" and he has it very much at heart to answer the sneer of the great London journal by proving from a survey of their work, that surgeons of Texas, country doctors though they be; though no long string of academic honors illustrate their names, are second to those of no country, in the variety, the boldness, and the success of operations; in practical skill, in fertility of resources, and in that self-reliance founded on knowledge, without which, no man can be a successful surgeon."

